# Diffuse Adrenal Gland and Pancreas Necrosis in a Patient with Disseminated Cryptococcosis—Case Report

**DOI:** 10.3390/life12101667

**Published:** 2022-10-21

**Authors:** Edina A. Wappler-Guzzetta, Austin L. Gray, Jessika Dagostino, Justin C. Kerstetter

**Affiliations:** 1 Department of Pathology, Loma Linda University Medical Center, Loma Linda, CA 92354, USA; 2 Pathologists’ Assistant Program, Loma Linda University, School of Medicine, Loma Linda, CA 92354, USA or; 3 Hoag Memorial Presbyterian Hospital, Newport Beach, CA 92663, USA

**Keywords:** disseminated cryptococcosis, necrotizing adrenalitis, necrotizing pancreatitis, case report

## Abstract

(1) Background: Cryptococcus neoformans is mostly known for causing meningitis, with or without disseminated disease. (2) Case presentation: An immunocompromised 75-year-old gentleman presented post renal transplant with generalized weakness, altered mental status, hypoxemia, and hyponatremia, and was found to have disseminated cryptococcal infection. After an initial improvement, the patient became suddenly hypotensive, and passed away soon after. The autopsy revealed widespread cryptococcal involvement, with the most severely affected organs being the brain, lungs, pancreas, adrenal glands, and spleen. The pancreas and one of the adrenal glands revealed diffuse granulomatous cryptococcal infection, with large areas of necrosis. The spleen also showed a large area of cryptococcal necrosis. In addition, the patient had chylous ascites, without histologically identifiable organisms. (3) Conclusions: This is a rare case of disseminated cryptococcal infection with severe necrotizing adrenalitis and pancreatitis, in addition to significant spleen, lung, and central nervous system involvement. The early recognition and treatment of the adrenal gland and pancreas cryptococcosis with surgical interventions may lead to better outcomes in affected patients. Furthermore, steroid treatment and diabetes mellitus may be risk factors for adrenal gland involvement. Additionally, clinicians should keep cryptococcal infection in their differential diagnosis for isolated adrenal gland and pancreas lesions.

## 1. Introduction

Cryptococcal infections most commonly cause meningoencephalitis and pneumonia in immunocompromised patients, including patients with HIV infection/AIDS, hematologic malignancies, severe liver disease, or patients on chemotherapy or immunosuppressant medications [1]. In addition, endogenous and exogenous hypercortisolism are also associated with a higher risk of certain fungal infections, including cryptococcosis [2,3]. Although the central nervous system (CNS) and lungs are the most common organs for cryptococcal involvement, other organs are frequently affected, especially in disseminated disease. Prior reports have shown involvement in the skin, liver, eye, lymph nodes (thoracic, and mesenteric most commonly), bone marrow, spleen, kidneys, prostate, thyroid gland, intestine, adrenal gland, pancreas, and ovaries by *Cryptococcus* [4,5]. Reports on pancreatic and adrenal gland infections are, however, rare, with sparse cases of severe organ damage. 

The purpose of this case report is to highlight the possible pancreatic and adrenal gland involvement with cryptococcal infection, which can significantly worsen the affected patient`s morbidity and mortality. Additionally, these cases might require surgical interventions in addition to anti-fungal therapy, in order to eradicate those foci of infection, ultimately improving outcomes.

## 2. Case Presentation

Here, we report the case of a 75-year-old gentleman who had a medical autopsy in our department. His past medical history was significant for type 2 diabetes mellitus (T2DM) and immunocompromised state following a cadaveric kidney transplant two years before his last hospital admission. His pre-admission medications included mycophenolate, tacrolimus, and prednisolone. He initially presented to the hospital with generalized weakness, altered mental status, hypoxemia, mild hyponatremia (130 mmol/L) and hypochloremia (96 mmol/L), and normal renal function (see admission blood results in Table S1), and he was treated for sepsis, requiring mechanical ventilation for a period of time. During the first few days of treatment his hyponatremia worsened (the lowest being 122 mmol/L), requiring tolvaptan treatment besides sodium chloride infusions. In regards to his immunosuppressive treatment, his tacrolimus was continued; however, mycophenolate was stopped. In addition, he received high-dose methylprednisolone injections during admission, followed by daily prednisolone treatment for the rest of his hospital stay. As his CSF and blood cultures were positive for *Cryptococcus neoformans* (*var grubii*), amphotericin and flucytosine were added to his treatment, which initially improved his condition, allowing for extubation. His mental status also significantly improved, with low/normal sodium levels (~135 mmol/L), and normal renal function. His clinical course later, however, was complicated by increased intracranial pressures, requiring intermittent cerebrospinal fluid drainage. Weeks later, he had a sudden deterioration with hypothermia, repeated hyponatremia (128 mmol/L), intermittent hypoglycemias (down to 50 mg/dL), altered mental status, and severe hypotension, requiring vasopressor therapy. This was followed by multiorgan failure, including renal failure, and despite all the treatment efforts, he passed away a few days after his deterioration.

**Figure 1:**
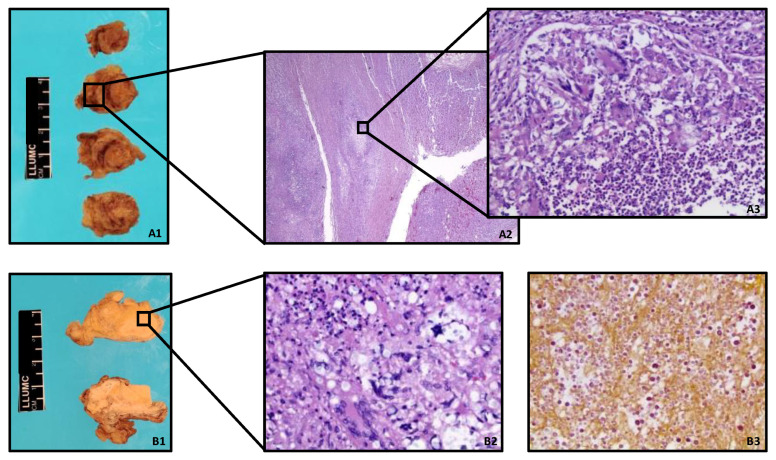
(**A1**): Gross image of one of the adrenal gland, showing multiple nodules involving the cortex and the medulla; (**A2**): microscopic image of the adrenal gland (H&E, 4×), with a necrotic inflammatory nodule; (**A3**): microscopic image of the adrenal gland (H&E, 40×), showing necrotizing adrenalitis with multiple giant cells and *Cryptococcal* organisms; (**B1**): Gross image of the pancreas with multiple tan nodules, involving the majority of the tissue; (**B2**): microscopic image of the pancreas (H&E, 40×), showing necrotizing pancreatitis with *Cryptococcal* organisms and occasional necrotizing granulomas. The pancreatic tissue is mostly taken over by necrosis and inflammation; (**B3**): *Cryptococcal* organisms are highlighted by mucicarmine stain (20×) in the pancreas tissue, consistent with *Cryptococcus*.

**Figure 2:**
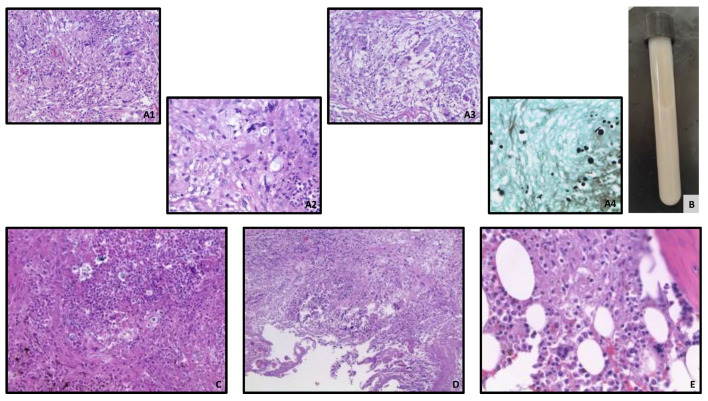
(**A**): Lung tissue ((**A1**): H&E, 20×; (**A2**): H&E, 40×) showing acute inflammatory reaction with numerous yeast-like organisms, highlighted by PAS ((**A3**), 20×; yeasts are magenta) and GMS stains ((**A4**), 40×; with yeasts staining black), consistent with fungal elements.; (**B**): Peritoneal fluid (**C**): Prostate tissue (H&E, 20×), showing acute prostatitis and the presence of yeasts; (**D**): Small bowel segment (H&E, 10×) showing acute inflammation and the presence of yeasts involving the muscularis layer; (**E**): Bone marrow (H&E, 40×) showing giant cells with occasional yeast elements.

His autopsy confirmed Cryptococcal meningoencephalitis and broncho-pneumonia. There were bilateral pleural effusions, and the lungs showed a significant, bilateral cryptococcal inflammation with foci of necrosis with a large number of foamy histiocytes and occasional giant cells, forming loose granulomas. Additionally, gross examination revealed multiple white-tan, firm nodules, involving the majority of one of the adrenal glands and the pancreas, which proved to be areas of cryptococcal necrosis and inflammation on histology (Figure 1). The pancreas and one of the adrenal glands showed widespread necrosis, with abundant fungi elements, surrounding by a zone of granulomatous inflammation and minute islands of normal tissue. The other adrenal gland showed only a small focus of destructive cryptococcal nodule in the cortex, consisting mostly of foamy macrophages. The spleen was enlarged with indistinct follicular markings on gross examination, and large areas of necrosis with abundant fungal elements on histology. The transplant kidney, the thyroid gland, and the bone marrow showed only small areas of inflammation, with the presence of occasional loose granulomas. In addition, significant, widespread cryptococcal prostatitis was seen, without stromal involvement. Yeasts were readily visible on most of the H&E slides in every organ, but were highlighted on selected tissue samples by Periodic acid–Schiff (PAS) and/or Grocott-Gomori’s (or Gömöri) methenamine silver stains. The fungi were mucicarmine-positive, consistent with *Cryptococci*. A significant amount of white-tan chylous fluid, with a distinct putrid odor, was also present throughout the peritoneal cavity. Surprisingly, cytologic examination of the fluid did not reveal definite cryptocococcal organisms. In addition, the serosal surface of the small bowel segment close to the pancreatic head had white-tan discoloration, showing cryptococcal involvement on histology. (Figure 2) The heart, testicles, liver, skin, and other parts of the gastrointestinal tract showed no signs of cryptococcal infection.

## 3. Discussion

Cryptococcus is a basidiomycete fungus disguised in a polysaccharide capsule, spreading via inhalation or skin inoculation with fungal cells from the environment. The human pathogen *Cryptococcus* species include *C. neoformans* (also known as *C. neoformans var grubii*), *C. deneoformans* (also known as *C. neoformans var neoformans*), and *C. gattii* species complex (also known as *C. neoformans var gattii*) [6]. While *C. neoformans* has a widespread distribution and is found in high concentrations in bird droppings and in the soil, *C. gattii* has been described in tropical and subtropical regions, and also in parts of Canada and the Pacific Northwest USA [5,6].

Although *C. neoformans* most often causes infection in immunocompromised patients, there are reports of cryptococcal infection in people with diabetes mellitus, autoimmune diseases, liver disease, and in seemingly healthy people too. *C. gattii*, on the other hand, often causes infection in immunocompetent hosts [5,7,8]. 

Cryptococcal infection typically causes granulomatous infection at the primary site of entry, which can be cleared or cause acute or chronic infection. Additionally, this facultative intracellular organism can survive in phagocyting cells, using them as “Trojan horses”. Disseminated disease can occur shortly after the primary infection or after a latency phase. During the latency period, the organism remains dormant, possibly for decades, and can be reactivated when the immune system weakens, for example during immunosuppressant therapy [6,7,9,10].

As previously mentioned, besides lung or CNS infection, *Cryptococci* frequently involve the eye, spleen, liver, bone marrow, kidney, prostate, lymph nodes, peritoneum, or skin [5,11,12]. Cryptococcal pancreatitis, however, is a rare entity, with only a few cases previously reported. The first reported case was a 59-year-old patient with Hodgkin lymphoma and disseminated cryptococcosis, who was found to have pancreatic involvement on her autopsy. The microscopic examination of her pancreas showed groups of *Cryptococci* without inflammation, along with occasional necrosis [13]. Another case presented in a pediatric acute-lymphoblastic leukemia patient, who was in remission when they suddenly died after developing severe abdominal pain. This case was proven to be cryptococcal infection on autopsy, where lung, heart, and pancreas involvement was described [14]. In addition, eight further cases of mostly asymptomatic pancreatic cryptococcal infections have been noted on autopsies in HIV-positive individuals [15]. Interestingly, a recent report describes another HIV-positive man who was found to have mesenteric lymphadenopathy and an ill-defined pancreatic tail mass on abdominal imaging, which latter proved to be a cryptococcal lesion [16]. Severe, multifocal pancreas necrosis, such as in the current report, has not been previously described. Clinicians, however, should have a suspicion for pancreas involvement in patients with cryptococcal infections with clinical signs or radiologic evidence of a pancreatic lesion, as this may significantly contribute to the patients’ morbidity and mortality. Surgical resection or endoscopic procedures might be necessary in selected cases, to improve the patient’s condition and to better control the infection.

The other unusual site of Cryptococcal infection in our case was the adrenal glands, where multiple small and large foci of necrosis were seen, both in the cortex and medulla. It is, however, more often seen than pancreatic involvement. In developing countries, the most common infectious causes of adrenal gland destruction are *Cytomegalovirus* and *Mycobacterium tuberculosis* (~10% of all cases), with fungal infection also rarely described. As opposed to *H. capsulatum* or paracoccidiomycosis, however, adrenal insufficiency is rare in other fungal infections, such as in cryptococcosis [17,18]. Independently of the cause, >90% of the adrenal gland must be unfunctional for most Addisonian symptoms to develop, with the majority being non-specific. These primary adrenal insufficiency symptoms include fatigue, anorexia, hypotension, hyperkalemia, hyponatremia, skin hyperpigmentation, and hypoglycemia, with sudden death also possibly being a presenting symptom [17,18,19]. In previous reports [5,19,20,21,22,23,24,25,26,27,28,29,30,31,32,33,34,35,36,37,38], adrenal gland involvement of cryptococci has been typically associated with disseminated disease, with CNS, or lung infections [5,20,21,22,23,24,25,26,27,30,32,33,36,37], but several exceptions exist in the literature (for complete list of previous cases with adrenal gland involvement see Table S2 [5,19,20,21,22,23,24,25,26,27,28,29,30,31,32,33,34,35,36,37,38]). These previous reports include patients with [23,24,31] or without an immunocompromised status [5,19,20,21,22,25,26,27,28,29,30,33,34,35,36,37,38], with several patients having T2DM as co-morbidity [5,28,30,35] or being on steroid treatment [21,36]. Symptomatic cases with large areas of adrenal gland necrosis are, however, sparse, with several cases needing unilateral- or bilateral adrenalectomies to control the infection [5,25,29,30,37]. In these cases, the infection was resistant to anti-fungal treatment alone. Sudden deterioration, such as in our patients’ case was also associated with large areas of adrenal gland necrosis in a previous case [20,21,25]. It is notable that several of these reports are on autopsy cases ([21,22,23,24,31,32], with 21–22 also being a case report), describing patients with or without HIV infection, and many showing only small areas of infection with or without inflammation. All these case and autopsy reports underline the importance of considering adrenal gland involvement in cryptococcal infections; and vice versa, to consider the possibility of cryptococcal infections in patients with adrenal lesions, to improve their outcome with adrenalectomy, if needed. It is also noteworthy that anti-fungal medications, such as azoles, can adversely affect adrenal function [3]; however, in our patient’s case, this was not the cause of adrenal insufficiency. Regarding our patient’s case, his sudden deterioration with severe hypotension and worsening hyponatremia and hypoglycemias weeks after his admission were most likely related to his widespread adrenal gland destruction. Unfortunately, at that point, adrenalectomy would not have changed his outcome, due to the severity of the widespread infection. It is unclear, however, if early detection—such as when he was hyponatremic on admission for a period of time—of the adrenal gland and pancreatic involvement could have better controlled the infection.

As to why *Cryptococci* have a tropism to lipids, such as the brain or possibly to the retroperitoneum, this is unclear. However, besides their main virulence factor, the large polysaccharide capsule, fungal metalloproteases are known to play an important role in CNS invasion [6,39]. Fungal lipid metabolism in general, and the presence of extracellular lipids and phospholipids are known to alter virulence in *Cryptococci* [6,40,41,42,43]. Additionally, host sphingolipids have been shown to alter granuloma formation and contribute to melanin production, and can alter microbial uptake via macrophages, phagocytic cell response, and neutrophil’s killing ability in Cryptococcal infection [43]. Besides, ferroptosis has been described in cryptococcal meningitis, involving rapid iron accumulation and lipid peroxidation [44]. Taken together, these may explain why *Cryptococci* have tropism to organs high in lipids, or with a significant amount of peri-organ fat; as in our patient’s case. It also highlights that these organs may serve as reservoirs for the fungus, enhancing cryptococcal infection and proliferation. Finally, *Cryptococcus* might be similar to *H. capsulatum* in its tropism to areas of high local concentrations of corticosteroids, such as the adrenal gland [3].

## 4. Conclusions

This is a case of disseminated cryptococcosis in an immunocompromised patient that was complicated by severe cryptococcal adrenalitis and pancreatitis, along with significant CNS, lung, prostate gland, and splenic involvement. The adrenal gland and pancreas involvement resulted in large areas of necrosis, which contributed to the patient`s morbidity, and likely to his mortality. Although these two organs are less known as involved in cryptococcal infections, this case highlights the need for clinicians to have a high index of suspicion for adrenal gland or pancreatic involvement in certain patient populations, such as patients on steroid treatments or patients with diabetes, as the symptoms can be non-specific or can be masked by severe illness. Cases of significant adrenal gland or pancreas involvements or in isolated infections may go unrecognized, allowing for significant morbidity before an appropriate treatment is initiated. Surgical intervention, in many of these cases, might alter the course of the disease.

## Data Availability

Not applicable.

## Supplementary Materials

The following supporting information can be downloaded at: https://www.mdpi.com/article/10.3390/life12101667/s1, Table S1: Patient`s laboratory data on day 1 of admission; Table S2: Clinical manifestations and organ involvement of previous cases of Cryptococcal adrenalitis.
