# Supplementary material for: Diffuse Adrenal Gland and Pancreas Necrosis in a Patient with Disseminated Cryptococcosis—Case Report
Source: Life (Basel). 2022 Oct 21;12(10):1667. doi: 10.3390/life12101667 (PMC9605411; doi:10.3390/life12101667)
Supplement: Supplementary file 1 [file life-12-01667-s001.zip › life-1960531-supplementary.pdf]

**Table S1.** Patient`s laboratory data on day 1 of admission

| Parameter | Patient`s value | Reference range | Units |
|---|---|---|---|
| **Na** | 130 | 135- 145 | mmol/L |
| **K** | 4.6 | 3.3- 5.3 | mmol/L |
| **Cl** | 96 | 98- 107 | mmol/L |
| **CO2 (venous)** | 28 | 22- 32 | mmol/L |
| **eGFR** | >60 | >60 | mL/min/1.73_m3 |
| **Blood urea nitrogen** | 17 | 5- 25 | mg/dL |
| **Creatinine** | 1.2 | 0.5- 1.4 | mg/dL |
| **Glucose** | 162 | 70- 110 | mg/dL |
| **Lactate** | 1.7 | 0- 2 | mg/dL |
| **Procalcitonin** | 1 | 0- 0.1 | μg/L |
| **WBC** | 6.9 | 4.5- 11 | x $10^9$/L |
| **RBC** | 5.16 | 4.5- 5.9 | x $10^{12}$/L |
| **Hgb** | 15 | 12- 16 | g/dL |
| **HCT** | 45.3 | 41- 52 | % |
| **MCV** | 87.8 | 80- 100 | fl |
| **MCHC** | 33.1 | 31.5- 36 | g/dL |
| **Platelet count** | 215 | 150- 450 | x $10^9$/L |

**Table S2.** Clinical manifestations and organ involvement of previous cases of Cryptococcal adrenalitis. Abbreviations: AF tx: anti-fungal therapy; AG: adrenal gland; AI: adrenal insufficiency; CNS: central nervous system; HT: hypertension; SAH: subarachnoid hemorrhage; Type-2 diabetes mellitus: T2DM; TB: tuberculosis; yo: -year-old; w: with; w/o: without.

| Clinical presentation | Immuno-competent | Co-morbidities | Organ involvement | Treatment | Outcome | Ref. |
|---|---|---|---|---|---|---|
| **A.** Autopsy report of a 41 yo patient w extensive fatigue, weakness, headache, uveitis, and no hypotension **B.** Plus: a review of further 7 cases from the literature (6 w/o symptoms of AI) | **A.** Yes **B.** Unknown | **A.** Chronic cough, headaches for 15 years, obesity **B.** TB, Hodgkin lymphoma, with others unknown | **A.** Adrenal glands (AGs) (with massive necrosis in all zones), meninges, kidneys, eye **B.** AGs (5:organisms w/o inflammation, 1: partial necrosis, 1: unknown) | **A.** Not treated/ not recognized before death **B.** Unknown | **A.** Patient suddenly deteriorated and died before the diagnosis was made **B.** Unknown | [20] |
| **A.** A case report of a 39 yo patient w frontal headaches, who developed meningitis and papilla edema, when found to have cryptococcal infection, and suddenly died on day 15 of | **A**. Yes, but on steroid treatment for co-morbidity **B**. Unknown | **A**. Temporal arteritis **B**. Unknown, but 18/41 patients were on steroid treatment | **A**. Complete AG destruction (with minimal normal tissue + large areas of necrosis in all zones), meninges, disseminated infection | **A.** Anti-fungal therapy (AF tx), and steroid (for his temporal arteritis) **B**. Unknown | **A.** The patient suddenly deteriorated and died **B.** N/A | [21] |

| | | | | | | |
|---|---|---|---|---|---|---|
| treatment with no other AI symptoms<br>**B.** Lookback on autopsy cases, with 11/41 showing AG involvement, 39 w disseminated cryptococcal infection | | | **B.** AGs (5/11 of cases with extensive involvement, but with large amount of normal tissue remaining; the rest w small lesions) | | | |
| 40 yo w persistent meningitis w unknown organism & years later w anorexia, weight loss, weakness, fatigue, hypotension, and hyponatremia | Yes | Unknown | AGs (w massive AG destruction, granulomatous inflammation & necrosis), disseminated infection | AF tx (diagnosis was made on resection specimen) | Improved | [22] |
| 3 of 15 patients on autopsy showed adrenal gland involvement | No | HIV | AGs, and disseminated infection | AF tx | N/A | [23] |
| 32 autopsy cases with presenting symptoms including vomiting, diarrhea, fever, hypotension, and hyponatremia; 3/41 with cryptococcal infection | No | HIV | 3/41 autopsy cases showed cryptococcal AG involvement, and disseminated infection | Unknown | N/A | [24] |
| 72 yo patient with progressive weakness, postural hypotension, anorexia, and weight loss | Yes | Not recorded | AG, meninges, lung | AF tx + bilateral adrenalectomy (massive caseation necrosis with fungal elements consistent with CN) | Patient deteriorated and died | [25] |
| 48 yo patient with fatigue, anorexia, weight loss, headaches, skin darkening, and acute right-sided weakness, hyponatremia | Yes | None | AGs,<br>Meninges | AF tx | Improved | [26] |
| 58 yo patient with AG enlargement and AI (confusion, weakness, anorexia, weight loss, increased skin pigmentation) | Yes | Previous SAH | AGs, CNS | AF tx | Unknown | [27] |
| 62 yo patient with flank pain | Yes | T2DM | AG | Diagnosis after surgical resection, AF tx started afterward | Successfully treated | [28] |
| 50 yo patient with anorexia, weight loss, nausea, increased skin pigmentation, | Yes | None | AGs initially;<br>Later with disseminated | Initially treated with anti-TB medications w/o evidence of TB infection; AF tx | Improved | [29] |

| | | | | | | |
|---|---|---|---|---|---|---|
| hyponatremia, and bilateral AG hyperplasia | | | disease involving the CNS | afterwards + bilateral adrenalectomy | | |
| 57 yo patient with headache and later developed hyponatremia | Yes | T2DM | AGs, Meninges | AF tx + bilateral adrenalectomy | Successfully treated | [30] |
| 7 of 128 autopsy cases showed cryptococcal, and 4 of 128 showed cryptococcal + CMV involvement in the AGs w inflammatory infiltrate and necrosis, rare fibrosis and hemorrhages | HIV infection | Unknown | AGs | Unknown | N/A | [31] |
| 1 of 13 autopsy cases showed AG involvement; and 45 yo patient with no clinical diagnosis of cryptococcal infection | Unknown | Meningitis in childhood, hydrocephalus, repeated drainage operations; and TB epinephritis on autopsy | AGs, spine | Not treated | N/A | [32] |
| 46 yo patient w primary AI | Yes | None | AGs | AF tx | Successfully treated | [19] |
| 45 yo patient w dizziness, fatigue, anorexia, weight loss, skin hyperpigmentation, hyponatremia, | Yes | None | AGs, disseminated infection | AF tx | No change in AG enlargement on imaging | [33] |
| 43 yo patient w high-grade fevers and weight loss | Yes | T2DM | AGs, Liver | AF tx + unilateral adrenalectomy | Significantly improved | [5] |
| 52 yo patient with weight loss and malaise for 3 weeks | Yes | Newly diagnosed mucinous adenocarcinoma, pT1b pN0 pMx, stage IA | AGs | AF tx, patient refused adrenalectomy when follow-up imaging showed no change in the size of adrenal nodules, but clinically improved | Successfully treated | [34] |
| 65 yo patient w fever | Yes | T2DM, HT | AGs, Lungs | AF tx | Successfully treated | [35] |
| 24 yo patient w fevers, headache, and altered consciousness | Yes, but on steroid treatment for co-morbidity | protein-losing gastro-enteropathy | AGs, Meninges | AF tx | Successfully treated | [36] |

| | | | | | | |
|---|---|---|---|---|---|---|
| 49 yo patient w 2 months history of anorexia, weight loss, and increased skin pigmentation | Yes | None | AGs, meninges | AF tx + bilateral adrenalectomy | resistant to prolonged AF tx but responding to bilateral adrenalecto my | [37] |
| 50 yo patient with AI, meningoencephalitis, and lytic bone lesions | Yes | None | AGs, CNS, skin, bone | AF tx | Successfully treated, but adrenal insufficiency remained | [38] |
